# Supplementary material for: A ribosomal kinetic checkpoint governs selective mRNA recruitment
Source: bioRxiv. 2026 Jun 13:2026.06.12.731988. Preprint. [Version 1] doi: 10.64898/2026.06.12.731988 (PMC13277876; doi:10.64898/2026.06.12.731988)
Supplement: Supplementary file 1 [file media-1.pdf]

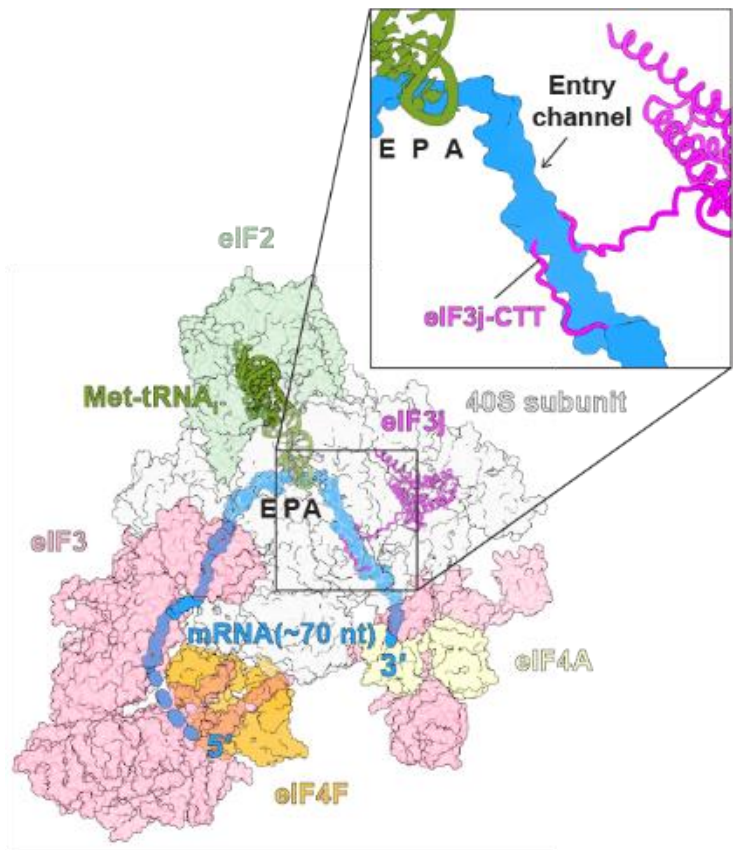

Figure S1. Structure of the human 48S complex.
The extended ~70 nt mRNA path is highlighted in blue (PDB 8OZ0[6]). The eIF3j (HCR1) C-terminal tail from the yeast 43S·ABCE1 structure (PDB 7A1G[13]) is superposed to highlight its position near the mRNA entry channel. The figure was created using ChimeraX[45].

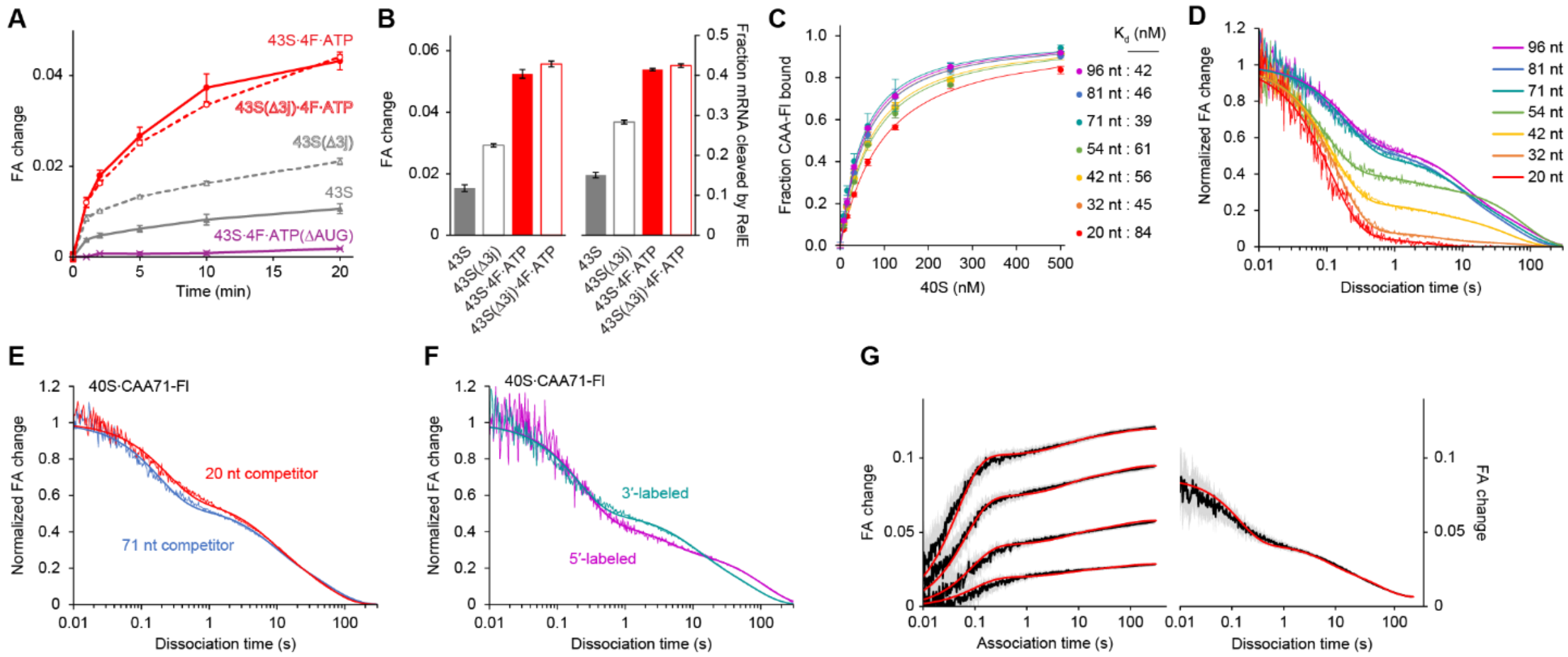

Figure S2. Kinetic analysis of mRNA binding to the 40S subunit.

(A) Start-site recognition kinetics monitored by FA changes of cap-CAA81(AUG)-Fl under conditions as indicated. **(B)** Comparison of end-point FA changes vs. RelE cleavages (after 40 min incubation). **(C)** Apparent affinities of 3′-labeled CAA repeats for the 40S subunit measured by equilibrium FA changes. **(D)** 40S dissociation kinetics for 3′-labeled CAA RNAs of varying lengths. **(E)** 40S dissociation kinetics for CAA71-Fl in the presence of excess unlabeled CAA71 or CAA20 competitor RNA. **(F)** 40S dissociation kinetics for CAA71 labeled at the 3′- or 5′-end. **(G)** Global fitting (red lines) for 40S·CAA71-Fl association and dissociation kinetic dataset, based on the branched model. Gray areas represent σ intervals for each curve. All data are represented as mean ± SEM (n=3).

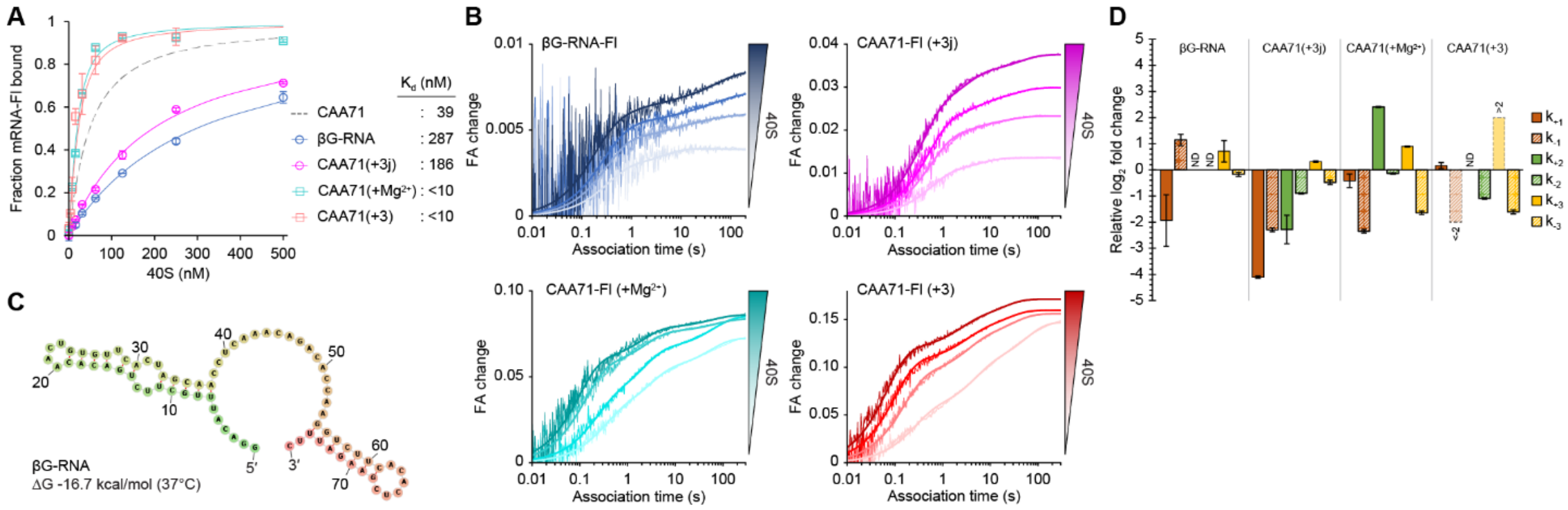

Figure S3. Kinetic details under various conditions.
(A) Apparent affinities of CAA71-Fl and βG-RNA-Fl for the 40S subunit under various conditions. **(B)** Association kinetics under various conditions. **(C)** Predicted secondary structure of βG-RNA using RNAfold web server[46]. **(D)** Relative $\log_2$ fold changes in kinetic rate constants under various conditions. 'ND' stands for not determined. Bars contoured with dashed lines represent estimates for a visual purpose. All data are represented as mean ± SEM (n=3).

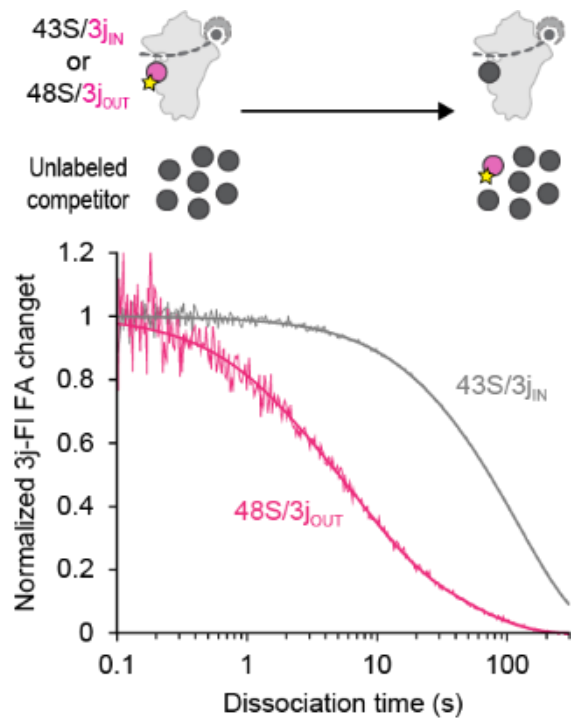

Figure S4. eIF3j dissociation kinetics for 43S/3j$_{IN}$ and 48S/3j$_{OUT}$.

Dissociation of 3j-Fl triggered by excess unlabeled competitor for the 43S (3j$_{IN}$ state) or the 48S (3j$_{OUT}$ state). Curves are fitted to double- or triple-exponential function as appropriate (thick lines). All data are represented as mean ± SEM (n=3).

Table 1. Kinetic and thermodynamic parameters of CAA-FI for the 40S binding

	CAA20	CAA32	CAA42	CAA54	CAA71	CAA81	CAA96
k_{-1} (s^{-1})	8.7 ± 0.5	7.0 ± 0.1	8.4 ± 0.4	10.5 ± 0.7	5.1 ± 0.2	5.6 ± 0.2	5.2 ± 0.4
k_{-2} (s^{-1})	0.45 ± 0.21	0.25 ± 0.03	0.34 ± 0.11	0.55 ± 0.11	0.11 ± 0.01	0.13 ± 0.01	0.11 ± 0.01
k_{-3} (s^{-1})	ND	0.012 ± 0.002	0.020 ± 0.001	0.012 ± 0.001	0.014 ± 0.001	0.017 ± 0.001	0.011 ± 0.001
A1	0.94 ± 0.011	0.91 ± 0.002	0.75 ± 0.005	0.59 ± 0.003	0.49 ± 0.001	0.46 ± 0.005	0.44 ± 0.008
A2	0.06 ± 0.011	0.05 ± 0.003	0.07 ± 0.005	0.08 ± 0.009	0.23 ± 0.002	0.26 ± 0.002	0.30 ± 0.005
A3	ND	0.03 ± 0.001	0.18 ± 0.004	0.34 ± 0.006	0.27 ± 0.002	0.28 ± 0.004	0.26 ± 0.007
$K_{d,app}$ (nM)	84 ± 14	45 ± 12	56 ± 6	61 ± 2	39 ± 10	46 ± 5	42 ± 0.5

*Values are mean ± SEM (n=3). ND stands for Not Determined.

Table 2. Kinetic and thermodynamic parameters of CAA71-Fl and βG-RNA-Fl for the 40S

	CAA71	Global fitting[a]	βG-RNA	CAA71+Mg^{2+}	CAA71+3j	CAA71+3
k_{-1} (s^{-1})[b]	5.1 ± 0.2	7.0	11.3 ± 2.4	1.0 ± 0.1	1.0 ± 0.1	ND
k_{-1}^{*} (s^{-1})[c]	6.1 ± 0.8		2.8 ± 0.3	1.9 ± 0.5	1.5 ± 0.2	<1
k_{-2} (s^{-1})[b]	0.11 ± 0.01	0.16	ND	0.10 ± 0.002	0.060 ± 0.001	0.052 ± 0.001[i]
k_{-3} (s^{-1})[b]	0.014 ± 0.001	0.014	0.010 ± 0.001	0.004 ± 0.001	0.010 ± 0.001	0.005 ± 0.001
k_{+1} (μMs^{-1})[c]	80 ± 5	68	21 ± 12	60 ± 10	4.6 ± 0.1	89 ± 7
k_{+2} (s^{-1})[d]	0.053 ± 0.005	0.055	ND	0.279 ± 0.004	0.011 ± 0.003	ND
k_{+3} (s^{-1})[d]	0.0077 ± 0.0007	0.0071	0.027 ± 0.008	0.030 ± 0.0004	0.020 ± 0.001	>0.04[i]
A1[e]	0.49 ± 0.001	0.54	0.31 ± 0.13	0.10 ± 0.003	0.31 ± 0.01	≤0.11[i]
A2[e]	0.23 ± 0.002	0.20	ND	0.26 ± 0.002	0.06 ± 0.02	≤0.11[i]
A3[e]	0.27 ± 0.002	0.26	0.69 ± 0.13	0.64 ± 0.005	0.63 ± 0.03	0.89 ± 0.02
$K_{d,app}$ (nM)[f]	39 ± 10		287 ± 13	<10	186 ± 6	<10
K_{d1obs} (nM)[g]	79		1202	ND	599	ND
K_{d1calc} (nM)[h]	65		538	17 ± 1	227	<10

[a]Values in this column were obtained by the global fitting
[b]Rate determined by the dissociation analysis.
[c]Rate determined by the observed association rate plot.
[d]Rate calculated according to the branched model: $k_{+2} = K2*k_{-2}$, $k_{+3} = K3*k_{-3}$, where K2 = A2/A1 and K3 = A3/A1
[e]Amplitude determined by the dissociation analysis.
[f]Apparent equilibrium dissociation constant determined by the experimental saturation curve.
[g]Dissociation constant according to $K_{d1obs}=K_{dapp}*(1+K2+K3)$
[h]Dissociation constant according to $K_{d1calc}=k_{-1}/k_{+1}$
[i]Estimated based on the first phase in dissociation kinetics (see Methods)
[*]Values are mean ± SEM (n=3). ND stands for Not Determined.

Table 3. Kinetic parameters of mRNA association with the 43S

	cap-CAA71				CAA71
	43S	43S·4F·ATP	43S·4F·ADP	43S·4F·ATP(ΔE)	43S·4F·ATP
k_{1obs} (s^{-1})[a]	1.30 ± 0.13	1.97 ± 0.08	1.65 ± 0.13	2.13 ± 0.32	2.75 ± 0.57
k_{2obs} (s^{-1})[a]	0.22 ± 0.03	0.43 ± 0.02	0.28 ± 0.04	0.41 ± 0.09	0.51 ± 0.04
k_{3obs} (s^{-1})[a]	0.037 ± 0.004	0.073 ± 0.005	0.051 ± 0.007	0.060 ± 0.006	0.086 ± 0.010
A_{1obs}[a]	0.39 ± 0.02	0.38 ± 0.03	0.43 ± 0.04	0.49 ± 0.05	0.37 ± 0.04
A_{2obs}[a]	0.40 ± 0.01	0.54 ± 0.02	0.43 ± 0.04	0.46 ± 0.05	0.55 ± 0.02
A_{3obs}[a]	0.20 ± 0.01	0.08 ± 0.01	0.14 ± 0.02	0.05 ± 0.01	0.08 ± 0.01
A_{2obs}/A_{3obs}[b]	1.00 ± 0.02	3.51 ± 0.15	1.59 ± 0.23	4.61 ± 0.65	3.65 ± 0.46
	cap-βG-RNA				βG-RNA
	43S	43S·4F·ATP	43S·4F·ADP	43S·4F·ATP(ΔE)	43S·4F·ATP
k_{1obs} (s^{-1})[a]	0.39 ± 0.11	1.35 ± 0.15	0.66 ± 0.06	0.70 ± 0.06	1.25 ± 0.22
k_{2obs} (s^{-1})[a]	0.034 ± 0.004	0.127 ± 0.002	0.056 ± 0.002	0.049 ± 0.001	0.127 ± 0.005
k_{3obs} (s^{-1})[a]	0.0072 ± 0.0005	0.0093 ± 0.0005	0.0094 ± 0.0005	0.0083 ± 0.0004	0.0085 ± 0.0003
A_{1obs}[a]	0.12 ± 0.01	0.13 ± 0.01	0.18 ± 0.01	0.11 ± 0.01	0.17 ± 0.01
A_{2obs}[a]	0.49 ± 0.02	0.65 ± 0.01	0.44 ± 0.01	0.56 ± 0.02	0.59 ± 0.02
A_{3obs}[a]	0.39 ± 0.03	0.23 ± 0.02	0.39 ± 0.01	0.33 ± 0.01	0.24 ± 0.02
A_{2obs}/A_{3obs}[b]	1.00 ± 0.15	2.28 ± 0.25	0.88 ± 0.03	1.35 ± 0.09	1.95 ± 0.19

[a]Observed rate or amplitude in association analysis
[b]A_{2obs}/A_{3obs} normalized to that for the 43S.
[*]Values are mean ± SEM (n=3).

Table 4. Kinetic parameters of eIF3j-Fl dissociation

Starting complex	43S·4F·ATP	43S·4F·ADP	43S·4F·ATP(Δ4E)
Triggered by	cap-CAA71	cap-CAA71	cap-CAA71
k_1 (s^{-1})	0.61 ± 0.03	0.61 ± 0.05	0.59 ± 0.01
k_2 (s^{-1})	0.061 ± 0.003	0.045 ± 0.015	0.061 ± 0.006
A_1	0.73 ± 0.01[a]	0.15 ± 0.01[a]	0.68 ± 0.01[a]
A_2	0.27 ± 0.01[a]	0.09 ± 0.02[a]	0.30 ± 0.01[a]
Δr[b]	0.043 ± 0.001	0.010 ± 0.001	0.042 ± 0.001
Starting complex	43S·4F·ATP	43S·4F·ADP	43S·4F·ATP(Δ4E)
Triggered by	cap-GlobinNluc	cap-GlobinNluc	cap-GlobinNluc
k_1 (s^{-1})	0.79 ± 0.16	0.32 ± 0.03	0.21 ± 0.03
k_2 (s^{-1})	0.0070 ± 0.0005	0.0048 ± 0.0014	0.0071 ± 0.0004
A_1	0.50 ± 0.01[a]	0.18 ± 0.03[a]	0.19 ± 0.02[a]
A_2	0.50 ± 0.01[a]	0.18 ± 0.02[a]	0.73 ± 0.11[a]
Δr[b]	0.013 ± 0.001	0.004 ± 0.001	0.012 ± 0.001
Starting complex	43S·4F cap-CAA71	43S	43S·4F·ATP cap-CAA71
Triggered by	ATP	Unlabeled eIF3j competitor	Unlabeled eIF3j competitor
k_1 (s^{-1})	0.79 ± 0.11	0.042 ± 0.004	0.73 ± 0.17
k_2 (s^{-1})	0.28 ± 0.03	0.0075 ± 0.0003	0.13 ± 0.01
k_3 (s^{-1})	0.022 ± 0.010		0.020 ± 0.002
A_1	-0.30 ± 0.11[c]	0.15 ± 0.02	0.26 ± 0.04
A_2	0.82 ± 0.04	0.85 ± 0.02	0.49 ± 0.03
A_3	0.18 ± 0.04		0.26 ± 0.01
Δr[b]	0.025 ± 0.004	0.104 ± 0.009	0.038 ± 0.002

[a]Amplitude normalized to the total anisotropy change for 43S·4F·ATP in the presence of the corresponding mRNA.
[b]Total reduction in eIF3j-Fl anisotropy.
[c]Negative value stands an increase in anisotropy.
*Values are mean ± SEM (n=3).

Table 5. List of mRNAs used in this study

Name	Sequence
CAA20	GGACAACAAAUGCAACAACA
CAA32	GGACAACAACAACAAACCCUCGAACAACAACA
CAA42	GGACAACAACAACAAACCCUCGAACAACAACAACAACAACAA
CAA54	GGACAACAACAACAAACCAUGGAACAACAACAACAACAACAACAACAACAACAA
CAA71	GGACAACAACAACAACAACAACAACAACAACAACAACAACAACAACAACAACAACAACA ACAACAACAACA
CAA81	GGACAACAACAACAACAACAACAACAACAACAACAACAACAACAACAACAACAACAACA ACAACAACAACAACAACAACAA
CAA81(AUG)	GGACAACAACAACAAACCCUCGAACAACAACAACAACAACAACACCAUGGACAACC AUGGCAACAACAACAACAACAA
CAA96	GGACAACAACAACAACAACAACAACAACAACAACAACAACAACAACAACAACAACAACA ACAACAACAAACCAUGGAACAACAACAACAACAA
βG-RNA	GGACAUUUGCUUCUGACACAACUGUGUUCACUAGCAACCUCAAACAGACACCAAGGUCU UCACACUCGAAGAUUUC
